# Self-care practices and associated factors among diabetes patients attending the outpatient department in Bahir Dar, Northwest Ethiopia

**DOI:** 10.1186/s13104-018-3874-8

**Published:** 2018-11-08

**Authors:** Teshager Weldegiorgis Abate, Minale Tareke, Mulat Tirfie

**Affiliations:** 1 Nursing Department, College of Medicine and Health Sciences, Bahir Dar University, P.O.BOX:79, Bahir Dar, Ethiopia; 2 Psychiatry Department, College of Medicine and Health Sciences, Bahir Dar University, Bahir Dar, Ethiopia; 3 Nutrition Department, College of Medicine and Health Sciences, Bahir Dar University, Bahir Dar, Ethiopia

**Keywords:** Self-care, Diabetes mellitus, Factors, Adult diabetes patient

## Abstract

**Objective:**

The aim of this study was to assess diabetes self-care practice and associated factors among diabetes patients attending Felege-Hiwot Referral Hospital, Bahir Dar, Northwest Ethiopia.

**Result:**

Prevalence of desirable self-care behaviors toward Diabetes Mellitus was 28.4% (95% CI 24.0–32.7%). There were significant association between the combined treatment modality of tablet with insulin (AOR: 2.72; 95% CI 1.01, 7.40), primary and secondary education level (AOR: 4.82; 95% CI 1.88, 12.35 and AOR: 3.08; 95% CI 1.26, 7.53, respectively). A considerable number of the patients had poor self-care practice, especially lack of social support and treatment modality, which have critical roles in controlling diabetes. Therefore, attention should be given to improve self-care practice.

## Introduction

Diabetes mellitus (DM) is a metabolic diseases characterized by hyperglycemia. It is a defect in insulin secretion, insulin action, or both [1]. DM is a global emergency: one death occurred every six seconds; one limb lost every 20 s [2]. More than 21 million people have DM in Africa and it will double by 2035. In Ethiopia, the expected number of diabetes was > 2.135 million [3]. DM was a cause of admission and boost disease-related complications [4]. Uncontrolled DM causes 2–4 times heart attacks, 2–6 times strokes, and 10–15 years shortens lifespan [5].

The goal of DM care is good metabolic control and render complications by the participation of health care professionals and client themselves [6, 7]. It required adequate self-care practices to keep the disease under control [8]. Self-care is self-limited, voluntary, and outside professional [9, 10]. In the previous studies, what was documented, high proportion of good practice of regular exercise (60%); planned dietary regimen (76%), taking medications (88.4%) [11, 12]. There was a poor self-care practice reported in the previous studies: poor regular exercising, regularly inspected and provided feet care [13–16].

Factors that redder self-care practice in DM patients were being male, housewife [17, 18], married, belonging to higher socioeconomic status, educational status [15–17, 19]; treatment modality [20, 21]. From all of the above review, studies available in the study area were limited in addressing factors that influence self-care practice. Therefore, this study aimed to assess self-care practices and associated factors among diabetes patients.

## Main text

### Methods

Institution—based cross sectional study was conducted from January 1st to April 8th, 2017 at FHRH. Currently, FHRH has provided promotive, preventive, curative and rehabilitative services. The service was rendered by physician and nurses. Patients with DM were visited the hospital on every 2 month basis. The sample size was determined by assuming 5% marginal error, 95% CI and 45% proportion of DM self-care practice **[**16**].** The final sample size including 10% non-response rate become 418 DM patients. All diabetes patients aged ≥ 18 years and who have been on regular follow for DM were included in this study.

Patients’ records were listed in follow up appointment order and used as a sampling frame. Then, Systematic random sampling was employed to select eligible participants. Based on the decision to collect data over the course of 1 month, sampling interval was determined by dividing the expected number of diabetic patients per month into the sample size which gives a sampling interval of three. Thus, every three-patient coming to a follow-up service was interviewed until the total sample size was then reached.

Trained four B.Sc. nurses collected data through face to-face interview and medical chart review. The questionnaire contains socio-demographic, economic, social support [22] and clinical factors. Clinical related factors like diabetes complication was gathered objectively by using checklists. Self-care practice patient with DM was evaluated by self-reported 18-item Summary of Diabetes Self-Care Activities (SDSCA) [23]. SDSCA scale measured frequency of self-care activity in the last 7 days; specifically general and specific diet, physical activity, blood glucose testing, foot care and medication. SDSCA was calculated by summing of the mean score for each dimension divided by the sum of the number of questions under each scale. After calculating an overall mean score, it was classified as having desirable self-care if scored ≥ 3 or not desirable self-care if scored < 3.

Structured questionnaire was prepared in English and translated into Amharic, then to check the consistency of the tool the Amharic version translated into English. The questionnaire was carefully evaluated and pre-tested on 5% of the sample size in patients with DM at Debretabor Hospital prior to actual data collection. The collected data entered into EPI—data version 3.1 and analyzed using SPSS version 20. Household wealth index was determined by using principal component analysis. The association was investigated by logistic regression. Variables with P-value < 0.2 in bi-variable analysis were selected for multivariable analysis. Finally, 95% CI and AOR were presented and interpreted.

### Results

Out of 418, 416 participants were interviewed making the response rate 99.5%. Of all participants 250 (60.1%) were married and 109 (26.2%) were farmers. The mean age (SD) of the respondents was 41.10 ± 15.65 years with minimum age 18 and maximum age 83 (Table 1).

**Table 1:** Socio-demographic characteristics of participant at FHRH, Bahir Dar, Northwest Ethiopia, 2017 (n = 416)

Variables	Category	Frequency	Percent
Sex	Male	240	57.5
	Female	176	42.3
Age	18–34	160	38.5
	35–44	73	17.5
	45–54	87	21.9
	≥ 55	96	23.1
Residency	Urban	173	41.6
	Rural	243	58.4
Marital status	Single	123	29.6
	Married	250	60.1
	Divorced	28	6.7
	Widowed	15	3.6
Educational status	Can’t read and write	133	32.0
	Read and write	70	16.8
	Primary school (1–8)	72	17.3
	Secondary school (9–12)	55	13.2
	College/University	86	20.7
Occupational	Farmer	109	26.2
	Civil servant	92	22.1
	Private worker	52	12.5
	Merchant	76	18.3
	House wife	59	14.2
	Others	28	6.7
Income	Very poor	83	20.0
	Poor	83	20.0
	Middle	84	20.2
	Rich	83	20.0
	Very rich	83	20.0

#### Clinical and psychosocial characteristics

The mean duration (SD) of diabetes treatment was 6.28 ± 4.87 years. Eighty-eight participants (21.2%) had diabetes retinopathy, which evidenced by a review of patients’ charts, and 209 (50.2%) had poor social support (Table 2).

**Table 2:** Clinical and psychosocial characteristics of participant at FHRH, Northwest Ethiopia, 2017 (n = 416)

Variables		Frequency	Percent
Types of diabetes mellitus			
Type 2		242	58.2
Type 1		174	41.8
Treatment modality			
Oral hypoglycemic with insulin		32	7.7
Only oral hypoglycemic		117	28.1
Only insulin		266	63.9
Only diet		6	1.3
Duration of DM (years)			
≤ 8		291	70.0
9–16		107	25.7
≥ 17		18	4.3
Taking another medication			
Yes		75	18.0
No		341	82.0
DM complication			
Diabetes retinopathy		88	21.2
Diabetes neuropathy		52	12.5
Sexual dysfunction		52	12.5
Foot ulcer		70	16.8
Social support			
Poor		209	50.2
Moderate		138	32.2
Strong		69	16.6

The overall prevalence of desirable self-care toward DM was 28.4% (95% CI 24.0–32.7%). DM patients who were taking a tablet combined with insulin had more likely to have a good self-care practice when compared to those other treatment regimes. Patients who were primary level education (AOR: 4.82; 95% CI 1.88, 12.35), widowed (AOR: 4.05; 95% CI 1.41, 11.58), and who have taken pill other medication (AOR: 2.87; 95% CI 1.43, 5.76) were more likely to have a good self-care practice. Whereas patients who were housewives (AOR: 0.34; 95% CI 0.13, 0.89) and who had poor social support (AOR: 0.29; 95% CI 0.15, 0.59) were more likely to have a poor self-care practice (Table 3).

**Table 3:** Predictors of self-care behavior among patients with diabetes at FHRH, Northwest Ethiopia, 2016 (n = 416)

Variable	Self-care practice(n)		COR (95% CI)	AOR (95% CI)
	Poor	Good		
Social support				
Strong	38	31	1	1
Moderate	97	41	0.52 (0.29, 0.94)	0.50 (0.25, 1.02)
Poor	163	46	0.35 (0.19, 0.62)	0.29 (0.15, 0.59)^a^
Educational				
Can’t read and write	109	24	0.54 (0.28, 1.02)	1.21 (0.46, 3.16)
Read and write	53	17	0.78 (0.38, 1.60)	1.60 (0.61, 4.23)
Primary school	41	31	1.85 (0.96, 3.57)	4.82 (1.88, 12.35)^a^
Secondary school	34	21	1.51 (0.74, 3.08)	3.08 (1.26, 7.53)^a^
College/University	61	25	1	1
Marital status				
Widowed	18	10	1.27 (0.56, 2.88)	4.05 (1.42, 11.58)^a^
Divorced	9	6	1.53 (0.53, 4.44)	1.86 (0.47, 7.39)
Single	98	26	0.61 (0.37, 1.02)	0.95 (0.49, 1.86)
Married	174	76	1	1
Occupational				
Civil servant	60	32	1	1
Farmer	89	20	0.42 (0.22, 0.80)	0.50 (0.19, 1.32)
Private worker	32	20	1.17 (0.57, 2.37)	0.95 (0.38, 2.34)
Merchant	50	26	0.97 (0.51, 1.84)	0.85 (0.36, 2.00)
House wife	47	12	0.48 (0.22, 1.03)	0.34 (0.13, 0.89)^a^
Others^b^	20	8	0.75 (0.29, 1.89)	0.55 (0.16, 1.82)
Income				
Very poor	73	10	0.25 (0.11, 0.56)	0.27 (0.12, 0.69)^a^
Poor	63	23	0.66 (0.34, 1.29)	0.77 (0.36, 1.68)
Middle	58	27	0.84 (0.44, 1.62)	0.96 (0.43, 2.10)
Rich	53	30	1.03 (0.54, 1.96)	1.13 (0.53, 2.42)
Very rich	51	28	1	1
Taking both oral and insulin				
Yes	16	282	2.77 (1.33, 5.73)	2.72 (1.01, 7.40)^a^
No	16	102	1	1
Taking other medication				
Yes	42	256	2.37 (1.41, 3.97)	2.87 (1.43, 5.76)
No	32	85	1	1

^a^Statically significant; *COR* crude odds ratio, *AOR* adjusted odd ratio

^b^Retires and students

### Discussion

In this study, good self-care practice among diabetes patients was 28.4% (95% CI: 24.0–32.7%) towards DM self-care practice in all domains. This level of good self-care practice was much lower than what has been documented in India, Harari, Addis Ababa and Iran [12, 16, 19, 20]. This might be due to a small sample size and difference setting used by the other study. The study conducted in India was small a sample size, whereas in our study relatively large; in Addis Ababa public hospital was a large sample size, whereas in our study smaller. It is much better when compared to what has been documented in other studies of India and Addis Ababa [18, 21].

In our study, those who under tablet combined with insulin therapy were 2.72 (95% CI 1.01, 7.40) more likely to have good self-care practice than those who were treated with other treatment modality. A contrasting result were found a study done in Iran 3.6 (95% CI 2.1, 5.7) and in Addis Ababa 1.94 (95% CI 1.31, 2.87) for those who under insulin treatment modality [19, 20]. Comparatively better self-care score among tablet with a combined of insulin therapy in the present study could be these people might have a diabetes which was uncontrolled by mono-therapy (tablets or insulin alone). The combined of insulin with a tablet therapy has complementary mechanisms of action [24, 25].

Social support was found to be significantly associated with good self-care practice. Similar findings were observed in a study done in Jimma [16], Addis Ababa [19] which showed that social support is important to ensure good self-care practice to the multiple diabetes related tasks among DM patients. Perhaps this might be due to effective social support may need to act as a more gentle guiding force that might be motivated behavioral change for the better self-care practice.

Our finding showed that income was one factor that affects self-care behavior in in diabetes patient. This result corresponds with the study finding in Harari [15], which observed a lower to good self-care practice among participants. This may be due to patients might be riskier life style with respect to income due to monetary condition, less accessibility and affordability of recommended self-care practice.

Our finding showed that participants who had formal education reported desirable self-care practice. This result corresponds to the study documented in Harari [15], Jimma [16] and Urban [20]. This might be a participant who had formal education might be escalated their base line information for self-care and could be successfully practiced self-care activities by reading guideline, and implement professional recommendations into practice.

In our study, participants those who were housewife less likely to have good self-care practice than those who were not; similar to the finding in India [18]. This might be different reasons, like easily appointed for carrying out the routine household work, probably lack information about self-care practice. An individual who had taken additional medication rather than a single dose of diabetes medication was one of the factors that affect self-care behavior in our study. This was not inline a study conducted in Harari [15]. In our study, taking addition medication might be consequence of drug–drug interactions, medication burden, adverse drug events, and functional decline [25].

Generally, this finding revealed that there were considerable numbers of participants had poor self-care practice (71.6%). Marital, economical and occupational status, educational level, taking additional medication, treatment modality and social support have a significant factor in order to provide patient own self-care. This suggests a health care providers aggressively emphasis on self-care practice by segmenting the patients based on social support, income, treatment regimens and educational status to motivate self-care practice.

## Limitation

Since the data were collected by health professionals working on follow up clinic there might be social desirability bias. The domain of self-care activities were obtained by self-reports and may be limited by recall bias. Another limitation of this study has not addressed whether participants have received any diabetes education or not.
